# Influenza virus and pneumococcal neuraminidases enhance catalysis by similar yet distinct sialic acid–binding strategies

**DOI:** 10.1016/j.jbc.2023.102891

**Published:** 2023-01-10

**Authors:** Laura Klenow, Rageia Elfageih, Jin Gao, Hongquan Wan, Stephen G. Withers, Jan-Willem de Gier, Robert Daniels

**Affiliations:** 1 Division of Viral Products, Center for Biologics Evaluation and Research, Food and Drug Administration, Silver Spring, Maryland, USA; 2 Department of Biochemistry and Biophysics, Stockholm University, Stockholm, Sweden; 3 Department of Chemistry, University of British Columbia, Vancouver, Canada

**Keywords:** sialidases, *N*-acetylneuraminic acid, influenza A virus, IAV, *Streptococcus* pneumoniae, mucosal pathogens, respiratory pathogens, BV, baculovirus, CBD, carbohydrate-binding domain, ELLA, enzyme-linked lectin assay, IAV, influenza A viruses, MUNANA, 4-methylumbelliferyl-α-D-*N*-acetylneuraminide, MWCO, molecular weight cut-off, SEC, size-exclusion chromatography, SEC-MALS, SEC with multiangle light scattering, TM, transmembrane, TEV, tobacco etch virus, TR1, titration reagent 1

## Abstract

Influenza A viruses and the bacterium *Streptococcus pneumoniae* (pneumococci) both express neuraminidases that catalyze release of sialic acid residues from oligosaccharides and glycoproteins. Although these respiratory pathogen neuraminidases function in a similar environment, it remains unclear if these enzymes use similar mechanisms for sialic acid cleavage. Here, we compared the enzymatic properties of neuraminidases from two influenza A subtypes (N1 and N2) and the pneumococcal strain TIGR4 (NanA, NanB, and NanC). Insect cell-produced N1 and N2 tetramers exhibited calcium-dependent activities and stabilities that varied with pH. In contrast, *E. coli*-produced NanA, NanB, and NanC were isolated as calcium insensitive monomers with stabilities that were more resistant to pH changes. Using a synthetic substrate (MUNANA), all neuraminidases showed similar pH optimums (pH 6–7) that were primarily defined by changes in catalytic rate rather than substrate binding affinity. Upon using a multivalent substrate (fetuin sialoglycans), much higher specific activities were observed for pneumococcal neuraminidases that contain an additional lectin domain. In virions, N1 and especially N2 also showed enhanced specific activity toward fetuin that was lost upon the addition of detergent, indicating the sialic acid–binding capacity of neighboring hemagglutinin molecules likely contributes to catalysis of natural multivalent substrates. These results demonstrate that influenza and pneumococcal neuraminidases have evolved similar yet distinct strategies to optimize their catalytic activity.

Neuraminidases are enzymes that catalyze hydrolysis of the glycosidic linkage that attaches terminal sialic acid residues to oligosaccharides, glycoproteins and glycolipids (1). This large family of sialidases are encoded by an array of organisms and several mucosal pathogens, including influenza viruses and the Gram-positive bacterium *Streptococcus pneumoniae* (pneumococcus), that target the human respiratory tract (2, 3). For influenza viruses, neuraminidase is a surface antigen that promotes penetration and viral movement in the respiratory mucosa by limiting the number of local sialic acid residues that can be bound by the more abundant hemagglutinin surface antigen (4, 5, 6). In contrast, pneumococcal neuraminidases have been shown to facilitate acquisition of sialic acid from the respiratory mucosa for use as a carbon source (7, 8, 9, 10) and to function as virulence factors in multiple models (11, 12, 13). Despite performing a similar function in the human respiratory tract, properties of influenza and pneumococcal neuraminidases have not been directly compared.

Influenza neuraminidases are Ca^2+^-dependent enzymes that function as tetramers on the viral surface (14, 15). The canonical six-bladed β-propeller structure that facilitates catalysis is located in the C-terminal head domain (16), and it is tethered to the viral surface by a variable length stalk region (17, 18, 19) and an amphipathic transmembrane (TM) helix (20). During synthesis neuraminidase inverts while it co-translationally translocates across the endoplasmic reticulum (ER) membrane, orienting the head domain in the ER lumen (21, 22, 23). With the help of resident ER chaperones, neuraminidases (24) assemble into tetramers by a cooperative process that involves both the TM and head domains (24, 25, 26). The folded tetramers are then trafficked through the *Golgi* to the plasma membrane for incorporation into budding virions.

Although nine different neuraminidase subtypes (N1-N9) are encoded by influenza A viruses (IAVs) from aquatic birds, only N1 and N2 subtypes are common in seasonal IAVs (*e.g.*, H1N1 and H3N2) that are endemic in humans (27). Independent of subtype, influenza neuraminidases generally exhibit optimal catalysis between pH 6 and 7 (28, 29), micromolar binding affinities for sialic acid (29, 30, 31, 32) and higher activity towards α2,3-linked sialic acids than α2,6-linked sialic acids (33, 34). Hydrolysis of sialic acid also occurs through a conserved mechanism that involves binding followed by the nucleophilic attack by an active site tyrosine at the sialic acid C-2 position, *via* an oxocarbenium ion-like transition state (35). The reaction cycle is completed by a nucleophilic attack of water at the anomeric center (C2) of the sialyl-enzyme intermediate resulting in sialic acid release *via* an oxocarbenium ion-like transition state.

Pneumococci commonly colonize the human nasopharynx and are one of the causative agents of secondary bacterial infections following influenza infection (36). Depending on the strain, pneumococci encode up to three neuraminidases (NanA, NanB, and NanC) with NanC being the least conserved with a prevalence of ∼50% across strains (37). Despite differences in sequence, they all possess a similar domain organization that includes an N-terminal signal peptide followed by a lectin or carbohydrate-binding domain (CBD) and a C-terminal catalytic domain that encodes for the canonical six-bladed β-propeller with the Asp boxes typical of bacterial neuraminidases (38). In the majority of strains NanA also contains an LPXTG motif that facilitates the sortase-mediated attachment to the cell wall; however, this motif is not present in the commonly studied TIGR4 strain (39).

All three pneumococcal neuraminidases fold following secretion from the cell and function as monomers. These enzymes have been reported to function in a wide pH range with optimums between pH 5 and 6.5 and to possess affinities for sialic acid that vary from micromolar to millimolar depending on the substrate (40, 41, 42). The highest affinity for sialic acid is attributed to NanA, which can cleave α2,3-, α2,6-, and α2,8-linked sialic acids, whereas the lower affinity NanB and NanC show cleavage specificity for α2,3-linked sialic acids (42, 43, 44). Sialic acid hydrolysis by the pneumococcal neuraminidases is thought to occur by a mechanism similar to the influenza neuraminidases, involving formation of a covalent sialyl enzyme intermediate followed by hydrolysis *via* an oxocarbenium ion-like transition state (35).

In this study, we produced recombinant versions of N1, N2, NanA, NanB, and NanC and performed a comparative analysis of their protein and enzymatic properties. Using the synthetic reporter substrate 4-methylumbelliferyl-α-D-*N*-acetylneuraminide (MUNANA), we demonstrated that catalytic activity rather than the substrate binding affinity defines the pH optimum for all the neuraminidases and that a recent N2 (2017 isolate) exhibits a stricter preference for pH 7. Changing to a multivalent sialoglycan substrate (fetuin) drastically altered the results. The differences were likely due to contributions from the lectin domain in the pneumococcal neuraminidases as NanB and NanC displayed higher specific activity on fetuin than N1 and N2 despite significantly lower sialic acid–binding affinities. The influenza neuraminidases, especially N2, also showed higher specific activity on fetuin in the context of virions which contain a high density of the sialic acid–binding protein HA. The concept of influenza neuraminidases using a spatially restricted lectin (HA) rather than a lectin domain to increase activity are discussed.

## Results

**Figure 1:**
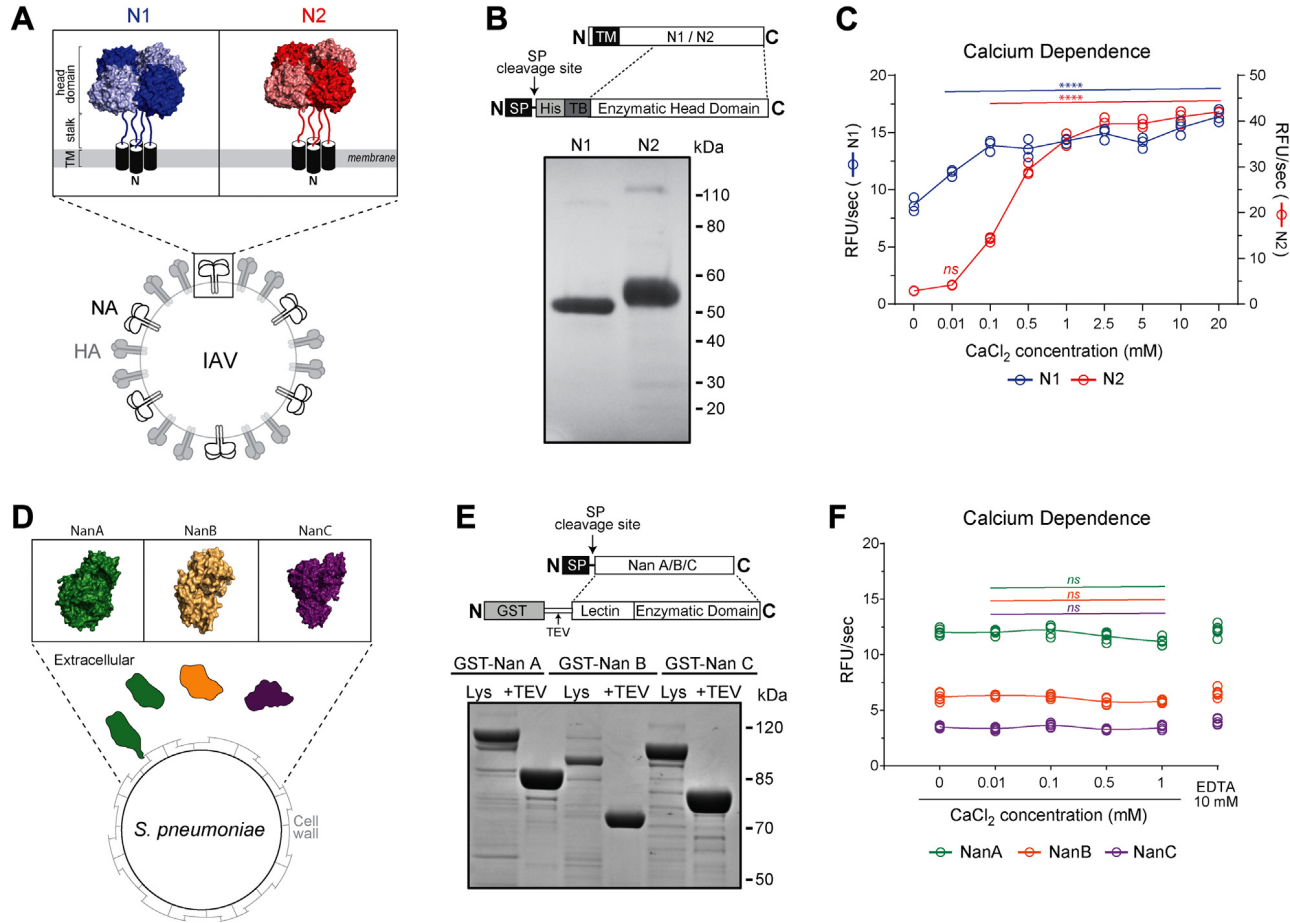
**Recombinant neuraminidase isolation and calcium dependence.***A*, diagram showing the topology and structure of neuraminidase subtypes 1 (N1) and 2 (N2) from seasonal (H1N1 and H3N2) IAVs. Structures of the tetrameric N1 (PDB ID: 3NSS) (76) and N2 (PDB ID: 4K1K) (77) head domains were generated with PyMol. *B*, recombinant N1 and N2 (∼5 μg each) were resolved under reducing conditions by SDS-PAGE (4–12% gel) and visualized by Coomassie staining. The N1 and N2 head domain constructs inserted into the baculovirus genome for insect cell expression are displayed above the gel. Regions corresponding to the transmembrane domain (TM), signal peptide (SP), 6× His tag (His), and the tetramerization domain (TB) are indicated. *C*, activity of the recombinant N1 and N2 in the presence of the indicated CaCl_2_ concentrations was determined using MUNANA. *p* values were calculated by a one-way ANOVA Dunnett’s multiple comparisons test with a 95% CI using 0 mM calcium as the comparison condition to characterize an increase in activity due to calcium addition. ∗∗∗∗*p* < 0.0001. *ns* – not significant. *D*, diagram showing the topology and structure of the three neuraminidases (NanA, NanB, and NanC) encoded by pneumococci. For NanA both the cell wall–associated and secreted variant are shown. PyMol was used to generate the NanA (PDB ID: 2YA4) (78), NanB (PDB ID: 2VW0) (43) and NanC (PDB ID: 4YZ1) (79) monomer structures. *E*, recombinant NanA, NanB, and NanC (∼5 μg each) were resolved under reducing conditions by SDS-PAGE (4–12% gel) and visualized by Coomassie staining. The constructs expressed in *E. coli* are displayed above the gel. Regions corresponding to glutathione S-transferase (GST), TEV protease site (TEV), and the signal peptide (SP) are indicated. *F*, recombinant NanA, NanB, and NanC activities in the presence of CaCl_2_ and EDTA were determined using MUNANA. IAV, influenza A viruses; MUNANA, 4-methylumbelliferyl-α-D-*N*-acetylneuraminide.

To examine the properties of neuraminidases from IAVs and *S. pneumoniae*, we generated recombinant N1 and N2 from two seasonal H1N1 and H3N2 vaccine strains and recombinant NanA, NanB, and NanC from the pneumococcal strain TIGR4. In IAVs, N1 and N2 tetramers are anchored to the viral envelop (membrane) by a stalk and an N-terminal TM helix (Fig. 1*A*). For production of soluble N1 and N2, the TM and stalk domains were replaced with a signal peptide followed by a 6× His tag and the tetrabrachion tetramerization domain (Fig. 1*B*). The latter compensates for the TM domain removal by stabilizing the enzymatic head domain in a tetrameric conformation (17, 45). The constructs were then introduced into baculovirus (BV) to facilitate high expression in Sf9 cells. The recombinant N1 and N2, isolated from the Sf9 cell medium, resolved at the expected molecular weight by SDS-PAGE and displayed ∼95% purity based on densitometry analysis (Fig. 1*B*). Enzymatic activity was confirmed with the synthetic substrate MUNANA, and calcium enhanced the activity of N2 more than N1 (Fig. 1*C*), supporting previous work on full-length N1 and N2 (14).

The three neuraminidases (NanA, NanB, and NanC) encoded by the pneumococcal strain TIGR4 possess an additional lectin domain and an N-terminal signal peptide (Fig. 1*D*). For recombinant protein production, the signal peptides from NanA, NanB, and NanC were replaced with a coding region for glutathione S-transferase and a tobacco etch virus (TEV) protease site. Each construct was then expressed in the *E. coli* cytoplasm, captured using glutathione resin and eluted by TEV cleavage (Fig. 1*E*). The eluted proteins resolved at the predicted molecular weights by SDS-PAGE, and the purity of each was greater than 85%. All three proteins were also confirmed to be enzymatically active using MUNANA (Fig. 1*F*). Neither calcium nor EDTA showed any effect, suggesting that NanA, NanB, and NanC have no accessible metal ions that are crucial for enzymatic activity.

**Figure 2:**
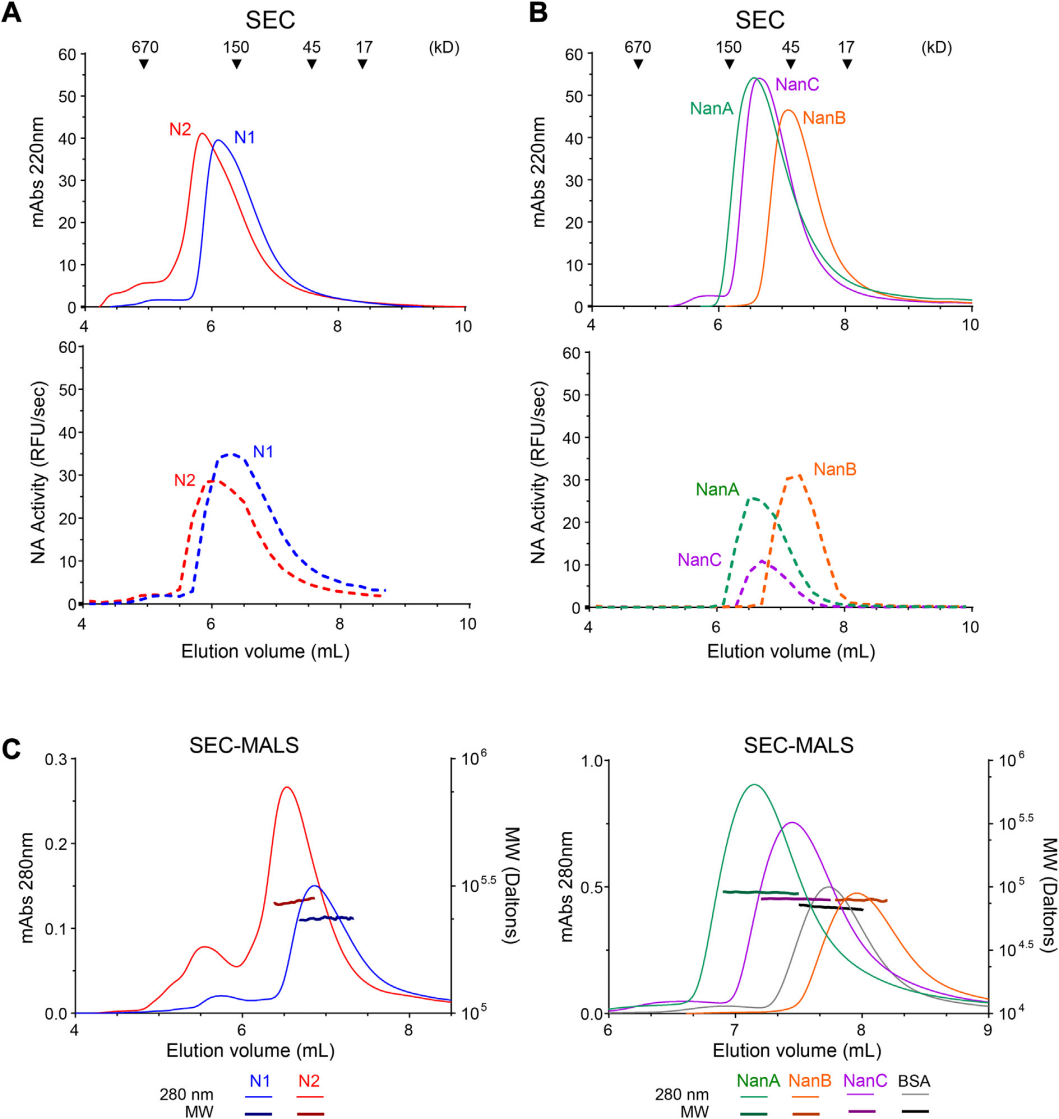
**Molecular weight and oligomeric state of the recombinant neuraminidases.***A*, graphs showing the size-exclusion chromatography (SEC) elution profiles of the (*upper panel*) recombinant N1 and N2 proteins and (*lower panel*) enzymatic activities. *Arrowheads* indicate the elution profile of the molecular weight standards. Activities are from equal volumes of the 200 μl fractions collected between 4 and 8.8 ml. *B*, graphs displaying the SEC elution profiles of the (*upper panel*) recombinant NanA, NanB, and NanC proteins and (*lower panel*) enzymatic activities. SEC elution profiles of the molecular weight standards (*arrowheads*) are depicted. Activities are from equal volumes of 200 μl fractions collected between 4 and 9.8 ml. *C*, SEC-MALS chromatograms of the (*left panel*) recombinant N1 and N2 and the (*right panel*) recombinant NanA, NanB, and NanC are displayed. For each protein the absorbance (Abs) reading at 280 nm is plotted along with the in-line molecular weight (MW) measurements obtained by multiangle light scattering (MALS).

The enzymatic activity results indicated that a portion of each recombinant protein preparation is functional and likely folded correctly. Further analysis by size-exclusion chromatography (SEC) showed that the recombinant N1 and N2 proteins are mainly homogeneous populations and that the proteins and activities elute at volumes consistent with a tetrameric conformation (Fig. 2*A*). NanA, NanB, and NanC also displayed homogeneous populations by SEC, and the proteins, as well as their activity, eluted at volumes indicative of a monomeric conformation (Fig. 2*B*). However, NanB eluted at a smaller size than what we expected based on the molecular weight of the protein and the standards, indicating NanB potentially interacts with the column stationary phase under our conditions. Therefore, we coupled SEC with multiangle light scattering (SEC-MALS) to directly measure the recombinant protein molecular weights. Supportive of being tetramers, the average molecular weight of the major N1 and N2 peaks by SEC-MALS were 236 kDa and 275 kDa, respectively (Fig. 2*C* left panel and Table 1). The bacterial neuraminidase molecular weights by SEC-MALS all showed good agreement with the expected monomeric molecular weights (Fig. 2*C* right panel and Table 1), indicating the delayed NanB elution profile, which came after the smaller 66 kDa control protein (BSA), is due to interactions with the column stationary phase.

**Table 1:** Neuraminidase molecular weights by SEC-MALS

Neuraminidase	Predicted monomeric molecular weight^a^	SEC-MALS molecular weight^b^
N1	51 kDa	236 ± 4.1 kDa
N2	52 kDa	275 ± 9.6 kDa
NanA	84 kDa	90 ± 2.2 kDa
NanB	75 kDa	79 ± 2.1 kDa
NanC	80 kDa	82 ± 2.5 kDa

a Predicted molecular weight is based on the amino acid sequence, excluding the N-linked glycans on N1 and N2.

b Average molecular weight ± range was calculated from the data shown in Figure 2*C*.

**Figure 3:**
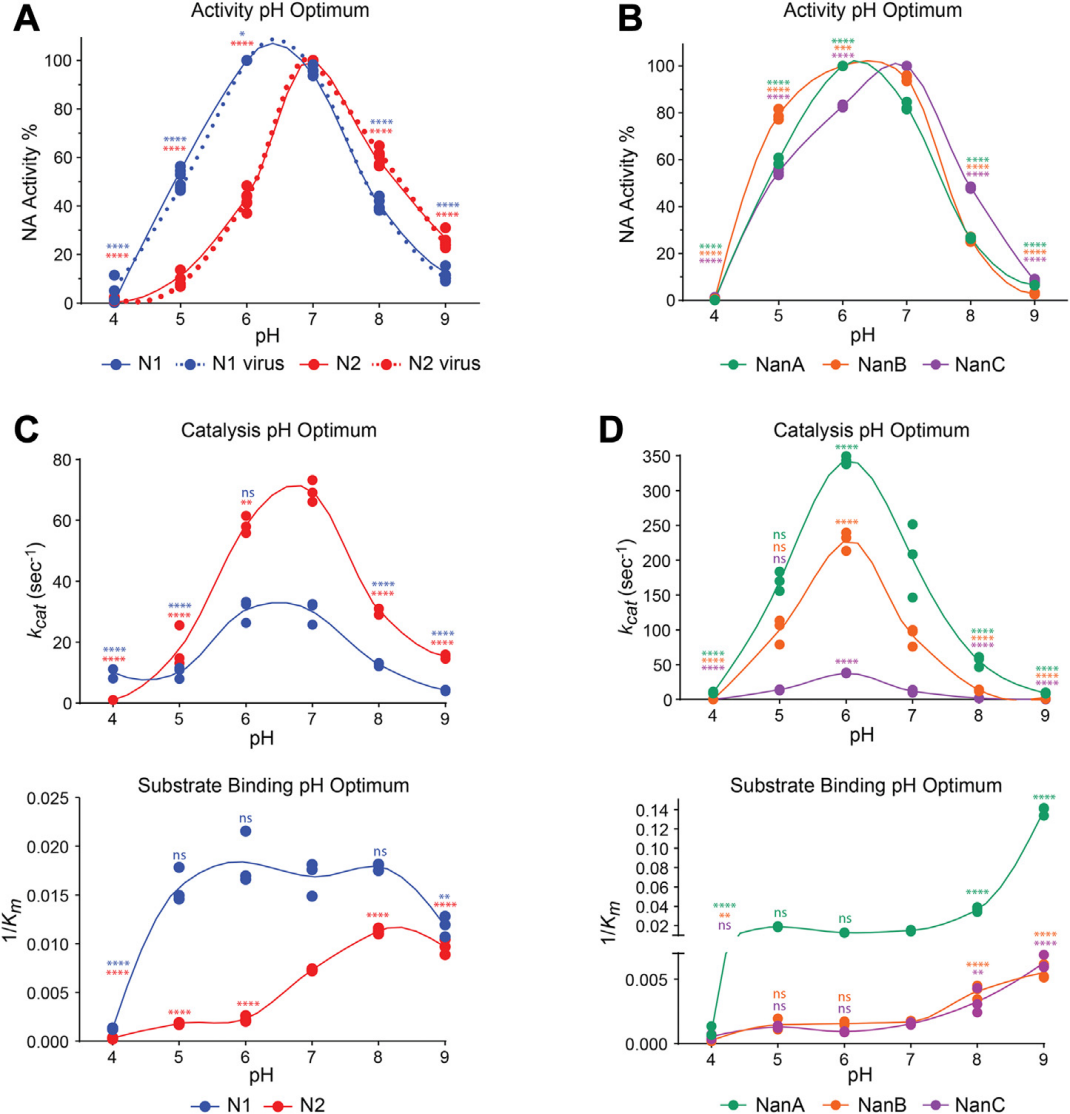
**Variation in neuraminidase activities by pH and the contributing properties.***A*, recombinant N1 and N2 activities were determined using buffers at the indicated pH values using MUNANA. The highest activity for each enzyme was set to 100% and used for normalization. Viruses containing identical full-length N1 and N2 were included as controls. *B*, recombinant NanA, NanB, and NanC activities were determined at the indicated pH values using MUNANA. The highest activity for each enzyme was set to 100% and used for normalization. *C*, graphs displaying the (*upper panel*) catalytic rate (*k*_*cat*_) and (*lower panel*) reciprocal Michaelis constant (*K*_*m*_) for N1 and N2 that were determined at each pH using MUNANA. *D*, NanA, NanB, and NanC (*upper panel*) *k*_*cats*_ and (*lower panel*) reciprocal *K*_*ms*_ were determined at each pH using MUNANA. *p* values were generated by a one-way ANOVA Dunnett’s multiple comparisons test with a 95% CI with pH 7 as the comparison condition to characterize an increase or decrease in activity due to changes away from neutral pH. ∗∗∗∗*p* ≤ 0.0001, ∗∗∗*p* ≤ 0.001, ∗∗*p* ≤ 0.01, ∗*p* ≤ 0.05, ns *p* > 0.05. MUNANA, 4-methylumbelliferyl-α-D-*N*-acetylneuraminide.

Neuraminidases from influenza and *S. pneumoniae* all function in the human respiratory mucosa which has been reported to vary from pH 5.6 to 6.7 in the nasal mucosa and increase up to pH 7 in the bronchia (46). Therefore, we examined if the neuraminidases possess different pH preferences for activity in this range. Interestingly, N1 displayed equally high activity at both pH 6 and 7, whereas N2 activity showed a distinct preference for pH 7 (Fig. 3*A*). Viruses carrying matching full-length N1 and N2 proteins yielded identical results, confirming the observed pH activity profiles are not unique to the recombinant proteins. Activity of the pneumococcal neuraminidases also peaked between pH 6 and 7 with NanA and NanB showing a slight preference for pH 6, whereas NanC displayed a slight bias for pH 7. Together these data support that the neuraminidases have evolved to function in the pH range of the respiratory tract mucosa.

To determine the contributions of catalytic activity and substrate binding to the pH optimum, we determined the *k*_*cat*_ and *K*_*m*_ for each protein at each pH using MUNANA. N1 catalytic activity was equally high at pH 6 and 7, whereas N2 catalytic activity was higher than N1 and showed a preference for pH 7 (Fig. 3*C*, upper panel, and Table 2). In the 1/*K*_*m*_ plot, a plateau was observed for N1 between pH 5 and 8 (Fig. 3*C*, lower panel, and Table 3), indicating N1 substrate binding is rather insensitive to pH changes and that the N1 pH optimum is mainly defined by changes in the catalytic rate. N2 substrate binding remained low from pH 4 to 6 and then dramatically increased at pH 7 until it peaked at pH 8 (Fig. 3*C*, lower panel and Table 3), implying the sharp N2 pH optimum results from a combination of the higher catalytic rate and the stronger substrate binding at pH 7 *versus* 6.

**Table 2:** Neuraminidase apparent and true catalytic rates for MUNANA in each pH buffer

pH	Apparent *k*_*cat*_*s*^*-1*^					True *k*_*cat*_*s*^*-1*^^a^	
	N1	N2	NanA	NanB	NanC	N1	N2
4	10 ± 2	1 ± 0	10 ± 1	0 ± 0	0 ± 0	53 ± 9	5 ± 0
5	10 ± 2	18 ± 7	170 ± 4	100 ± 18	14 ± 1	54 ± 10	77 ± 29
6	31 ± 4	58 ± 3	344 ± 6	228 ± 13	38 ± 1	163 ± 20	253 ± 12
7	30 ± 4	69 ± 4	202 ± 53	91 ± 13	12 ± 2	160 ± 20	300 ± 16
8	13 ± 1	30 ± 1	55 ± 8	13 ± 1	2 ± 0	67 ± 3	132 ± 5
9	4 ± 0	15 ± 1	9 ± 1	1 ± 0	0 ± 0	22 ± 2	66 ± 3

a Values were adjusted based on the fraction of active N1 and N2 in preparations measured by TR1.

**Table 3:** Neuraminidase Michaelis constants (*K*_*m*_) for MUNANA in each pH buffer

pH	N1 (μM)	N2 (μM)	NanA (μM)	NanB (μM)	NanC (μM)
4	767 ± 56	3471 ± 1082	1255 ± 445	4299 ± 1046	2044 ± 794
5	64 ± 7	558 ± 33	54 ± 2	724 ± 199	778 ± 58
6	55 ± 8	444 ± 56	79 ± 2	646 ± 59	1074 ± 63
7	60 ± 7	137 ± 2	67 ± 4	593± 25	652 ± 37
8	56 ± 1	88 ± 2	27 ± 2	250 ± 36	324 ± 91
9	85 ± 8	104 ± 8	7 ± 0	183 ± 18	160 ± 13

In contrast, catalytic activity for all the pneumococcal neuraminidases showed a clear preference for pH 6 with NanA exhibiting the highest catalytic rate followed by NanB and NanC (Fig. 3*D*, upper panel, and Table 2). With respect to substrate binding, the pneumococcal neuraminidases all displayed a plateau in the 1/*K*_*m*_ plot between pH 5 and 7, which then increased until the last measurement at pH 9 (Fig. 3*D*, lower panel, and Table 3). Together these results imply that the pH optimums for NanA, NanB, and NanC are primarily defined by changes in the catalytic rate and that the subtle shift toward pH 7 for NanC is due to a small substrate binding increase that counteracts the decrease in catalytic activity.

**Figure 4:**
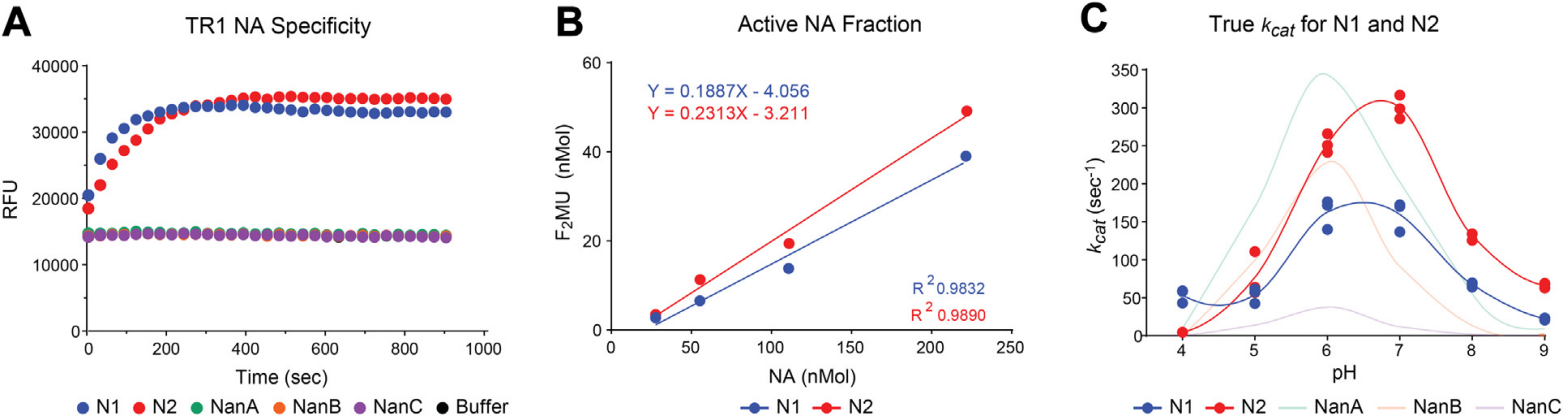
**Determination of true catalytic rates for recombinant N1 and N2.***A*, graph displaying the reactivity of recombinant N1, N2, NanA, NanB, and NanC with the influenza neuraminidase titration reagent TR1. *B*, correlation plot showing the number of F_2_Mu molecules that were produced following incubation of TR1 with increasing numbers of N1 and N2 molecules. The slope of the linear fit corresponds to the fraction of active N1 and N2 molecules in each recombinant preparation. *C*, the theoretical N1 and N2 *k*_*cats*_ at each pH (Fig. 3*C*, *upper panel*) were adjusted for the fraction of enzymatically active molecules to determine the true *k*_*cat*_ values. NanA, NanB, and NanC, theoretical *k*_*cats*_ (Fig. 3*D*, *upper panel*) were included for reference. TR1, titration reagent 1.

To measure the catalytic rates more accurately, we used the influenza neuraminidase titration reagent 1 (TR1) to quantify the number of functional active sites in each protein preparation (47, 48). In line with the specificity for influenza neuraminidases, TR1 reacted with N1 and N2 but not with the pneumococcal neuraminidases (Fig. 4*A*). TR1 was then incubated with N1 and N2 and the concentration of F_2_MU that was produced was plotted with respect to the N1 and N2 concentrations to determine the proportion of enzymatically active molecules. Consistent with a recent study (49), ∼19% of the N1 and ∼23% of the N2 in the recombinant protein preparations were found to be enzymatically active (Fig. 4*B*). Using these values, the theoretical *k*_*cat*_ values for N1 and N2 were adjusted and found to be more similar to NanB and NanA than NanC (Fig. 4*C*, and Table 2), assuming the full-length recombinant pneumococcal neuraminidases in the preparations are almost 100% active.

**Figure 5:**
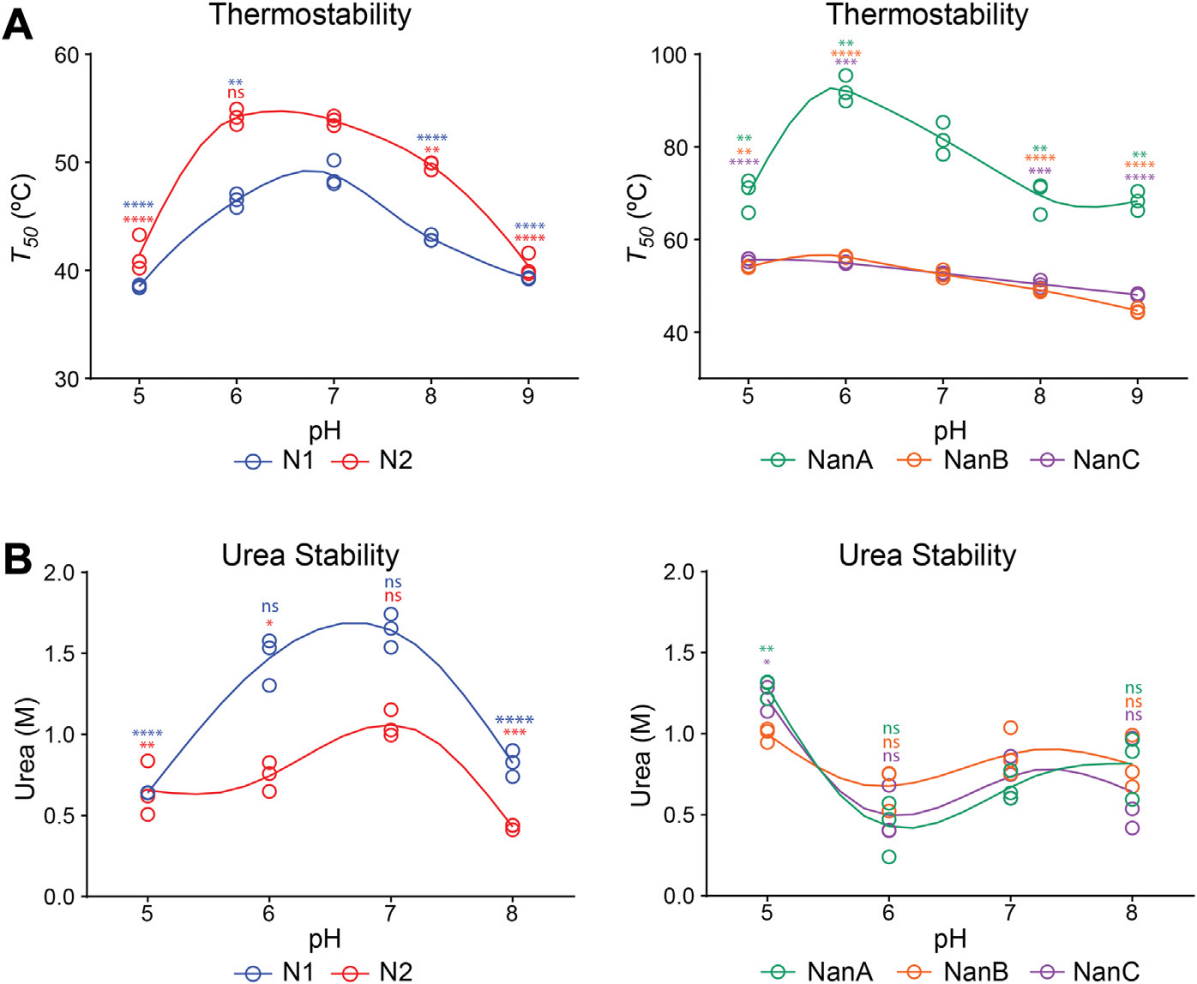
**Stability variation between the neuraminidases.***A*, thermostability of (*left panel*) N1 and N2 and (*right panel*) NanA, NanB, and NanC was determined at each pH by performing a melt curve analysis that used enzymatic activity as a readout and calculating the *T*_*50*_. Data for each protein are from three independent biological replicates. *B*, urea stability of (*left panel*) N1 and N2 and (*right panel*) NanA, NanB, and NanC was assessed by measuring the enzymatic activity at each pH in the presence of increasing concentrations of urea in three independent experiments. The urea concentration corresponding to 50% inhibition of activity is displayed from each experiment. *p* values were generated using a one-way ANOVA Dunnett’s multiple comparisons test with a 95% CI, using pH 7 as the comparator. ∗∗∗∗*p* ≤ 0.0001, ∗∗∗*p* ≤ 0.001, ∗∗*p* ≤ 0.01, ∗*p* ≤ 0.05, ns *p* > 0.05.

Influenza neuraminidases are known to display low thermostability that is calcium dependent (14, 50). To determine if N1 and N2 thermostability varies with pH, we diluted N1 and N2 in different pH buffers and performed a melt curve analysis to calculate the *T*_*50*_, which corresponds to the temperature where 50% of the original activity remains. N2 generally displayed higher thermostability than N1 and both showed the highest stability at pH 7 with N2 displaying equal stability at pH 6 (Fig. 5*A*, left panel). A similar analysis revealed that the pneumococcal neuraminidase thermostability is generally higher and that large pH-dependent stability changes were only observed for NanA, which showed a bias for pH 6 (Fig. 5*A* right panel). As a complement, we also performed activity melt curves using increasing concentrations of the denaturant urea. Interestingly, N1 was more resistant to urea denaturation than N2 and both displayed the highest denaturation resistance at pH values that coincided with optimal activity, pH 6 and 7 for N1 and pH 7 for N2 (Fig. 5*B*, left panel). In contrast, urea denaturation profiles for the pneumococcal neuraminidases were similar with a slight increase in resistance at pH 5 (Fig. 5*B* right panel). The steady stability observed for the monomeric pneumococcal neuraminidases suggests that N1 and N2 stability is more sensitive to pH changes due to the functional requirement of being a tetramer.

**Figure 6:**
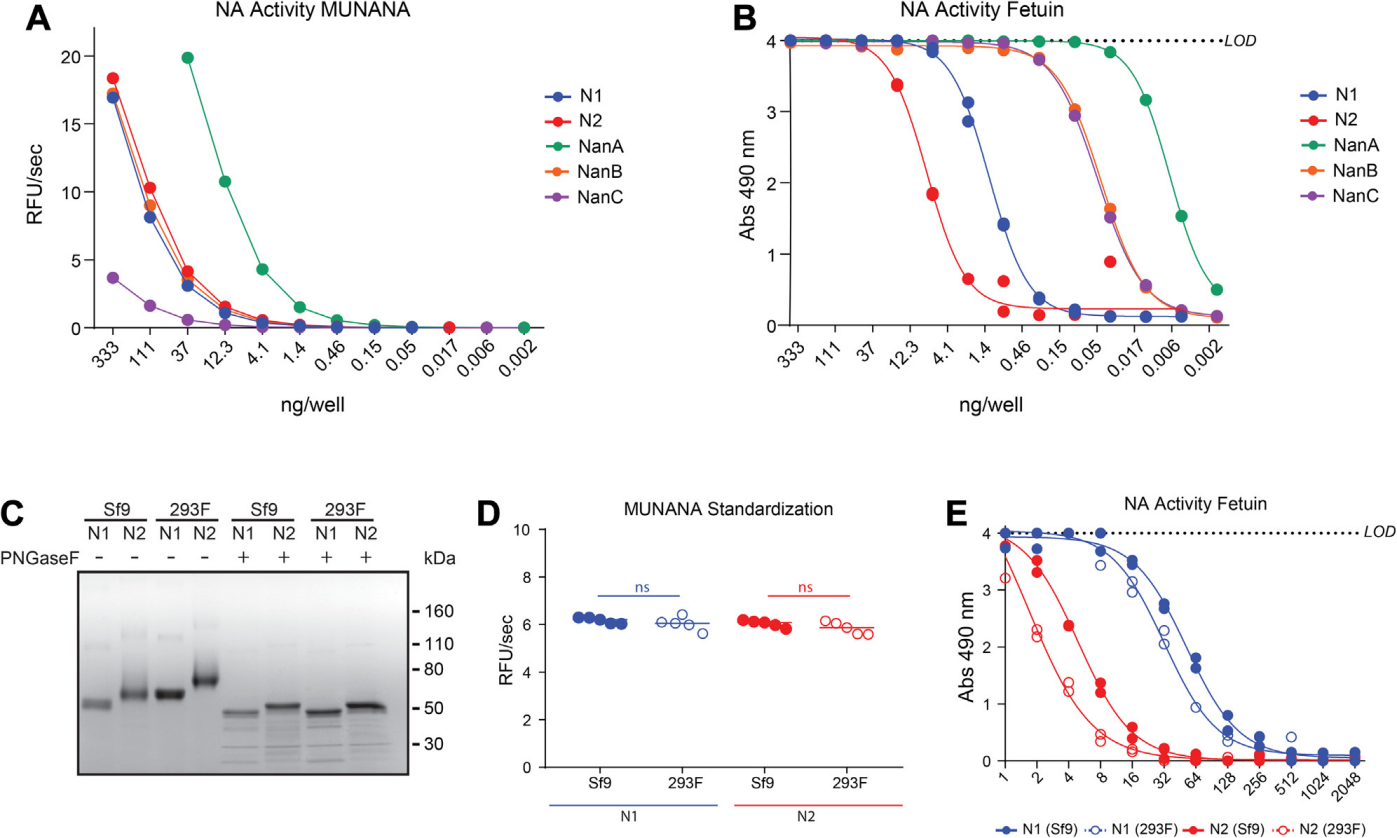
**Comparison of the neuraminidase activities using a biologically relevant substrate.***A*, specific activities of each recombinant neuraminidase were determined in the pH 6.5 ELLA buffer using MUNANA. *B*, graph displaying the specific activities of the recombinant proteins that were measured by an ELLA using the sialoglycans from immobilized bovine fetuin as a substrate. *C*, Coomassie-stained SDS-PAGE gel of recombinant N1 and N2 proteins produced in either insect (Sf9) or mammalian (293F) cells. Proteins (∼2 μg) were treated with or without PNGaseF to remove *N-*linked glycans prior to resolution under reducing conditions. *D*, recombinant N1 and N2 proteins were standardized based on activity that was measured using MUNANA. *p* values were generated with a two-tailed unpaired *t* test (95% CI). ns *p* > 0.05. *E*, the MUNANA standardized samples (from Fig. 6*D*) were serially diluted across a plate containing immobilized bovine fetuin, and the enzymatic activity was measured by an ELLA. MUNANA, 4-methylumbelliferyl-α-D-*N*-acetylneuraminide; ELLA, enzyme-linked lectin assay.

To analyze the properties of the neuraminidases on a more natural multivalent substrate, we used fetuin, which is known to contain mono-antennary, bi-antennary, and tri-antennary glycans with terminal α2,3- and α2,6-linked sialic acids (51) and compared the results to MUNANA. With MUNANA as a substrate the specific activity of NanA was the highest, NanC showed the least specific activity and NanB, N1 and N2 were all found to possess similar specific activity (Fig. 6*A*). However, when bovine fetuin glycans were used as a substrate, the specific activity changed dramatically (Fig. 6*B*). NanA remained the highest, followed by NanB and NanC, which were indistinguishable, and then N1 and N2, suggesting the pneumococcal neuraminidase CBDs likely increase their apparent catalytic rates of sialic acid hydrolysis on natural substrates such as a glycoprotein where the glycans are sterically linked.

IAVs generally replicate in avian or mammalian cells that add larger more complex *N*-linked glycans than insect cells (52). To examine if the *N*-linked glycan composition alters the N1- or N2-specific activity on fetuin glycans, we produced the same constructs in mammalian 293F cells and used PNGaseF to confirm that the proteins possess larger *N*-linked glycans than their insect cell counterparts (Fig. 6*C*). We then standardized the recombinant proteins by activity using MUNANA (Fig. 6*D*) and tested the specific activity on fetuin (Fig. 6*E*). The N1 and N2 proteins produced using insect cells both displayed slightly higher specific activities (less than two-fold) than the 293F expressed versions, indicating the *N-*linked glycan composition on N1 or N2 does not dramatically impact the enzymatic activities on a multivalent substrate.

**Figure 7:**
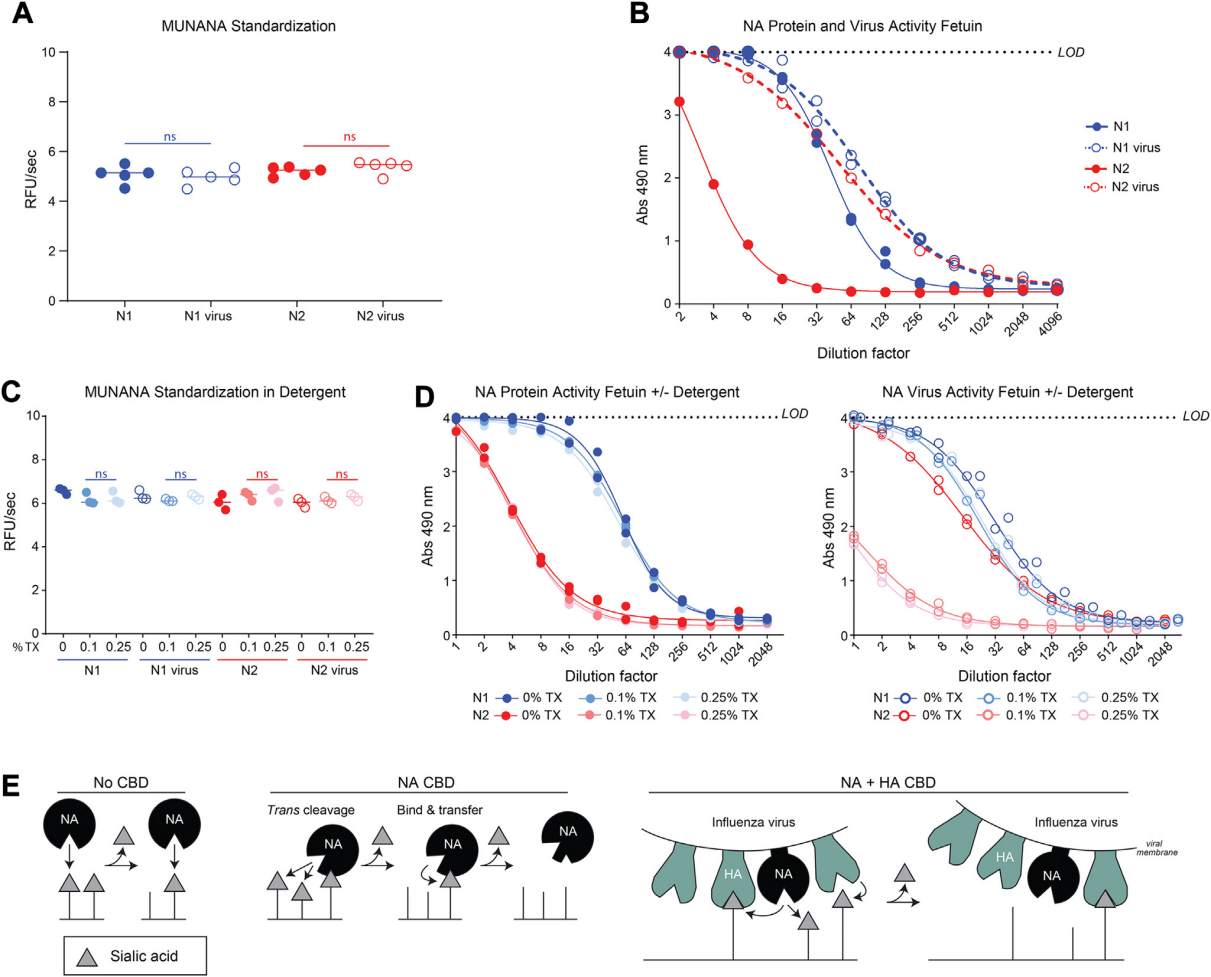
**Virions alter influenza neuraminidase activity on a biologically relevant substrate.***A*, recombinant N1 and N2 were standardized to influenza viruses carrying matching N1 and N2 sequences based on activity that was measured using MUNANA. *p* values were generated with a two-tailed unpaired *t* test (95% CI). ns *p* > 0.05. *B*, the MUNANA standardized samples (from Fig. 7*A*) were serially diluted across a plate containing immobilized bovine fetuin, and the enzymatic activity was measured by an ELLA. *C*, standardized recombinant N1 and N2 protein and viruses were treated with the indicated concentrations of Triton X-100 (TX), and the activities were measured using MUNANA. *p* values (95% CI) were determined by a one-way ANOVA using the sample lacking TX as the comparator. ns *p* > 0.05. *D*, the recombinant N1 and N2 protein (*left panel*) and virus (*right panel*) samples (from Fig. 7*C*) that possessed equal activity against MUNANA were serially diluted across a plate containing immobilized bovine fetuin in buffer containing the indicated TX concentrations. Enzymatic activities were measured by an ELLA. *E*, a schematic displaying how neuraminidase (NA) activity on multivalent substrates varies in the absence (No CBD) and presence of a CBD (NA CBD) and how HA can act as a CBD surrogate for influenza neuraminidases in virions (NA + HA CBD). MUNANA, 4-methylumbelliferyl-α-D-*N*-acetylneuraminide; ELLA, enzyme-linked lectin assay; CBD, carbohydrate-binding domain.

Based on the lower specific activity of the recombinant N1 and N2 proteins toward fetuin, we hypothesized that in a virus the sialic acid–binding capacity of HA could perform a similar role to the pneumococcal neuraminidase CBDs. To test our hypothesis, we standardized the soluble recombinant N1 and N2 proteins to viruses carrying the same N1 and N2 by activity using MUNANA (Fig. 7*A*) and tested the specific activity on fetuin (Fig. 7*B*). Strikingly, the N2 specific activity increased ∼10-fold when it was in a virion carrying an HA that has been shown to efficiently bind the sialoglycans on bovine fetuin (53). In contrast, N1 displayed only a subtle increase in specific activity (less than two fold) when it was in a virion with an identical HA, suggesting the higher N1 sialic acid–binding affinity, or the existence of a secondary sialic acid–binding site (54), limit the contribution of HA sialic acid binding to the apparent N1 activity on a multivalent substrate.

To confirm the specific activity increase was more likely attributed to the presence of HA rather than the membrane integrated form of full-length N1 or N2 produced in eggs, we used detergent to disrupt the virion and separate HA from N1 or N2. Treatment with Triton X-100 (0.1% or 0.25%) did not significantly alter any of the activities measured by MUNANA (Fig. 7*C*), and it also did not change the recombinant N1 and N2 specific activity on fetuin (Fig. 7*D*, left panel). However, detergent treatment of the viruses appeared to remove the HA contribution as the N1 and N2 specific activities on fetuin were reduced to levels more in line with the recombinant proteins with N2 showing a much more dramatic reduction (Fig. 7*D*, right panel). Together, these results demonstrate that influenza and pneumococcal neuraminidases both increase sialic acid cleavage of natural multivalent substrates by utilizing additional CBDs. For influenza neuraminidases, the additional CBD comes in the form of the spatially restricted neighboring HA proteins on the virion surface, and the contribution from HA is likely to vary based on the inherent properties of different N1 and N2 variants.

## Discussion

Pneumococci are one of the major causative agents of secondary bacterial pneumonia that can develop following an influenza virus infection (36). In addition to functioning in the same environment, the neuraminidases from these two respiratory pathogens have been shown to contribute to this lethal synergy in animal models (3, 9, 55, 56). Based on this link, we compared the properties of an N1 and an N2 from two recent influenza vaccine strains (A/Brisbane/02/2018 (H1N1) and A/Kansas/14/2017 (H3N2)) and NanA, NanB, and NanC from a pneumococcal strain (TIGR4). Our results show that influenza neuraminidase activity increases upon Ca^2+^ addition and that the stability varies with pH, whereas pneumococcal neuraminidases are insensitive to Ca^2+^ and EDTA and possess robust stability. In line with the pH of the human respiratory tract, (57) all the neuraminidases displayed optimal activity between pH 6 and 7, and this was mainly determined by changes in the catalytic rate rather than substrate binding. With a multivalent substrate, pneumococcal neuraminidases exhibited much higher specific activities, indicating that the additional lectin domains enhance the activity on biological substrates. Virions containing N1 and especially N2 also showed higher specific activity toward the multivalent substrate, suggesting that sialic acid binding by neighboring HA molecules performs a similar function to the pneumococcal neuraminidase lectin domain.

Two models have been proposed for how a CBD can contribute to catalysis of multivalent substrates (54, 58, 59) and we modified these to account for the contribution of HA in a virion (Fig. 7*E*). For neuraminidases that lack a second sialic acid–binding site (No CBD), substrate binding is primarily driven by the active site affinity for sialic acid, and hydrolysis of the glycosidic bond causes dissociation from the multivalent substrate. In contrast, neuraminidases that possess a second sialic acid–binding site (NA CBD) are thought to function either by a *trans-*cleavage or a bind and transfer model. In the *trans-*cleavage model, the enzyme remains bound to a multivalent substrate *via* the CBD while it cleaves neighboring sialic acid molecules, whereas in the other model, the CBD directly binds the substrate before transferring it to the active site for cleavage. In virions that contain HA (NA + HA CBD), both mechanisms can occur simultaneously without dissociation from the multivalent substrate due to the presence of multiple HA molecules that are physically connected by the viral membrane.

Neither model for how a CBD contributes to catalysis is mutually exclusive, both increase the apparent sialic acid concentration for the enzyme, and the difference is largely dependent on the sialic acid–binding affinity of the second site *versus* the catalytic site with the latter being lower for N2 than N1. For NanB and NanC, the specific activity on the multivalent substrate fetuin was much higher than N1 and N2 despite the lower sialic acid–binding affinity of the enzymatic domain. Based on these data and the nonuniform sialic acid concentration in our immobilized fetuin assay (60), we speculate the NanB and NanC lectin domains function in the targeting *trans*-cleavage model, increasing the local sialic acid concentration to compensate for the low binding affinity. Supporting this theory, the NanB and NanC lectin domains likely possess functional and structural similarities to the NanA lectin domain which has been solved bound to sialic acid with ∼2 μM affinity for both α2,3- and α2,6-linked sialic acids (61, 62), which is much higher than the enzymatic domain affinities we measured.

In virions, influenza neuraminidases are spatially restricted to an environment that is crowded with sialic acid-binding HA molecules. Individually, HA molecules have a low (millimolar) sialic acid–binding affinity, but in a virion, HAs can form polyvalent interactions which can increase the apparent affinity multiple orders of magnitude (63). These properties support both models as the HAs can target virions containing neuraminidase to the substrate and the dissociation by the individual HAs can allow for cleavage of the sialic acid. Thus, it was not surprising that N2 showed a large specific activity increase with the multivalent substrate when it was in a virion. This result implies that the activity of at least this N2 on multivalent biological substrates is highly HA dependent, which makes it easy for the virus to maintain the necessary functional ‘balance’ between NA and HA as the NA catalysis mainly occurs following HA binding.

Interestingly, N1 specific activity only showed a modest increase with the multivalent substrate when it was in a virion, suggesting the benefit from HA sialic acid binding is significantly reduced compared to the N2 we examined. One contributing factor is likely the higher sialic acid–binding affinity of the enzymatic domain and another is that the viruses possessed different HAs and likely different N1 and N2 content. An additional factor could be the presence of a second sialic acid–binding site on the enzyme that would be expected to function much like the lectin domain on the pneumococcal neuraminidases. Shallow second sialic acid–binding sites have been reported on N1, N2, N5, N6, and N9 (64, 65, 66, 67). These sites are comprised of the residues from three surface loops (370, 400, and 430) and are highly conserved in these neuraminidase subtypes from avian IAVs, but less so in human IAVs (54). Indeed, prior work has shown that a weak second sialic acid–binding site is present in recent related human N1s that contributes to the activity on a multivalent substrate (68). Thus, it is likely that the combination of the second sialic acid–binding site and the higher affinity for sialic acid reduce the contribution of HA to recent N1 activity on multivalent substrates.

Our results also have implications for maintaining the conformation of recombinant influenza neuraminidase vaccine antigens during purification (*e.g.*, buffer compositions) and for the enzyme-linked lectin assay (ELLA) used for evaluating influenza neuraminidase antigenicity (60, 69). The ELLA for comparing antigenicity is identical to the fetuin assay from this study, but it generally uses viruses as the neuraminidase source, and the evaluation is determined by comparing the ability of different specific antisera to inhibit cleavage of the sialoglycans on fetuin. From our data, it is clear the antigenicity assay is HA-dependent, and the dependence varies with the properties of the neuraminidase, suggesting an HA-independent neuraminidase antigenicity assay needs to be developed to avoid any potential HA interference in the readout, especially from HAs that are different. One possibility would be to use recombinant protein, but this assumes the recombinant neuraminidases will all fold properly, and it potentially puts too much weight on the second sialic acid–binding sites that are likely compensated for in virions containing HA. It also is not clear how relevant the results from recombinant neuraminidases are to full-length neuraminidases in virions.

It has been reported that the human nasal mucosa is approximately pH 6 and that it increases to pH 7 in the bronchia (46). Therefore, it was intriguing that N2 showed a stricter activity preference for pH 7, whereas N1 activity was similar at both pH 6 and 7. Although our data show this was due to a unique pH-dependent increase in substrate binding affinity, it is not clear if this property is a more general trend for N2 or strain specific. In addition, sialic acid modifications such as N-acetylneuraminic acid and N-glycolylneuraminic acid have different impacts on IAVs (70). While bovine fetuin has been reported to possess small amounts of N-glycolylneuraminic acid (71), it is not clear how the neuraminidase activities or the relationship between influenza neuraminidase and HA binding on multivalent substrates would vary based on the ratios of these two main species of sialic acid. Future studies using larger panels of substrates together with neuraminidases from avian, swine, and human influenza strains will help to provide more insight about whether these properties are human-related adaptations or potential compensations for changes in the properties of the HA with which it is paired.

## Experimental procedures

### Reagents

Rosetta 2 cells and SEC molecular weight standard were from Agilent. Imidazole, isopropyl β-D-1-thiogalactopyranoside (IPTG), 1 ml HisTrap Fast Flow crude columns, glutathione-sepharose, Tris, phosphate, citric acid, CaCl_2_, protease inhibitor cocktail (PIN), Sf-900 III serum free medium (Sf-900), FreeStyle 293 (293F) cells, FreeStyle 293 expression media, simple blue stain, Novex WedgeWell 4 to 12% Tris-Glycine SDS-PAGE gels, Novex sharp unstained protein standard, Maxisorp 96-well plates, and BCA protein assay kit were obtained from Thermo Fisher Scientific. Centrifugal filters (10 and 30 kDa), polyethylenimine, and 4-methylumbelliferyl-α-D-*N*-acetylneuraminide (MUNANA) were acquired from Amicon, Polysciences, and Cayman Chemical, respectively. Bovine fetuin, 4-methylumbelliferone sodium salt (4-MU), o-phenylenediamine dihydrochloride, valproic acid, and HRP-linked peanut agglutinin were purchased from Sigma. Low protein binding 96-well black clear bottom fluorescent assay plates were from Corning. Specific-pathogen-free hen’s eggs were purchased from Charles River Labs. The reassortant Influenza A viruses WSN^H1N1/BR18^ (N1 virus), which carries the HA and NA genes from the strain A/Brisbane/02/2018 (H1N1), and WSN^N2-KS17^ (N2 virus), which carries the N2 gene from the strain A/Kansas/14/2017, were generated for prior studies (53, 72).

### N1 and N2 recombinant protein production

pFastBac1 and pcDNA3.1 vectors containing the N1 and N2 protein constructs were synthesized by Genscript and used to generate recombinant BVs or recombinant protein directly. The N1 pFastBac1 construct encodes the acidic glycoprotein GP67a signal peptide followed by a 6× His-tag, tetrabrachion tetramerization domain (residues 1238–1286 (73)), thrombin cleavage site, 7 residue linker, and N1 residues 82 to 469 from the H1N1 strain A/Brisbane/02/2018 (49). N2 was designed similarly using residues 74 to 469 from the H3N2 strain A/Kansas/14/2017. Except for a CD5 signal peptide, the pcDNA3.1 constructs were identical. For insect cell protein expression, Sf9 cells were grown at 27.5 °C in Sf-900 medium in a dark shaking (125 rpm) incubator to a density of 2 to 4 × 10^6^ cells/ml with ≥ 95% viability prior to infecting with a 2% v/v recombinant N1 or N2 BV stock (passage 3). For mammalian protein expression, 293F cells were grown in a humidified 37 °C, 8% CO_2_ incubator on a rotating platform (135 rpm) using shake flasks and 293F expression medium. Prior to transfection, cells were sedimented (220*g*; 5 min) and resuspended to 2 × 10^6^ cell/ml with prewarmed medium that contained the NA expression plasmid at a final concentration of 2 μg/ml, and the cells were returned to the incubator for 5 min. Polyethylenimine was diluted with prewarmed medium and was added to the culture (final concentration 9 μg/ml). At 24 h posttransfection, cells were diluted 1:1 with prewarmed medium containing valproic acid at a concentration of 5 nM.

### N1 and N2 recombinant protein production and purification

The same procedure was used for both proteins and cell lines. At 72 to 96 h post-infection (insect cells) or post-transfection (293F cells) cultures were harvested, sedimented (10 min; 10,000*g*), supernatants were decanted and concentrated 7 times by tangential flow filtration with a 30 kDa molecular weight cut-off (MWCO) capsule (Pall) followed by 5 volumes of diafiltration into Binding buffer (50 mM Tris pH 7.5, 150 mM NaCl, 1 mM CaCl_2_, 40 mM imidazole). N1 and N2 were then purified by immobilized metal affinity chromatography using an AKTA Start equipped with a 1 ml HisTrap column. After loading the samples, the column was washed with 10 column volumes (CVs) Binding buffer and the bound protein was eluted with a 20 CV linear imidazole gradient (40 mM to 500 mM). Fractions (1 ml) with high NA activity were pooled, buffer exchanged to 50 mM Tris pH 6.5, 150 mM NaCl, 1 mM CaCl_2_ using 30 kDa MWCO centrifugal filters and the protein concentration was determined by a BCA Protein Assay Kit and adjusted to ∼0.5 mg/ml prior to aliquoting and storing at −80 °C. Densitometry analysis of Coomassie stained SDS-PAGE gels was performed using ImageJ to determine purity.

### Generation of NanA, NanB, and NanC expression plasmids

Coding regions for mature NanA (nucleotides 1598071–1595828), NanB (nucleotides 1589152–1587143), and NanC (nucleotides 1251553–1249409) were amplified from *S. pneumoniae* TIGR4 genomic DNA (GenBank ID: AE005672.3) by PCR and cloned into a modified pGEX-6P-1 plasmid (Sigma) that contained a TEV Protease site in place of the PreScission Protease site. The resulting expression plasmids encoding glutathione-S-transferase linked to a TEV protease site, and NanA, NanB, or NanC were all verified by sequencing (Eurofins).

### TEV protease expression and purification

Recombinant TEV protease was produced in *E. coli* Rosetta 2 cells using a pMal plasmid encoding Maltose binding protein fused to TEV protease and a C-terminal 6× His tag which was kindly provided by David Drew (Stockholm University). Three freshly transformed Rosetta 2 cell colonies were grown in a shaking (200 rpm) incubator at 37 °C in 300 ml Lysogen broth containing 100 μg/ml carbenicillin. At an *A*_600_ ∼0.6 (600 nm), the culture was induced (0.05 mM IPTG) and moved to a room temperature shaking (200 rpm) incubator for 16 h. Cells were sedimented (10 min; 10,000*g*), and the pellet was resuspended in 30 ml lysis buffer (20 mM Tris, pH 7.5, 300 mM NaCl, 20 mM imidazole, 1 mM DTT, 10 mg/ml lysozyme, 1 mM MgCl_2_, 1× PIN, and a speck of DNAse I) and rotated 1 h at 4 °C. Cells were sonicated on ice (three 20 s intervals at 10% amplitude), sedimented (30 min; 50,000*g*) at 4 °C. The supernatant was passed over a 1 ml HisTrap column using an AKTA Start followed by 20 CVs of buffer (20 mM Tris, pH 7.5, 300 mM NaCl, 20 mM imidazole) and 10 CVs of elution buffer (20 mM Tris, pH 7.5, 300 mM NaCl, 250 mM imidazole). Fractions (1 ml) containing TEV protease were identified using Coomassie stained SDS-PAGE gels, pooled, dialyzed (10 kDa MWCO) twice against 1 L of buffer (20 mM Tris pH 7.5, 300 mM NaCl, and 1 mM DTT), and adjusted to 1 mg/ml with 50% glycerol and stored at −80 °C.

### NanA, NanB, and NanC recombinant protein production and purification

For each construct, three freshly transformed Rosetta 2 cell colonies were used to inoculate 300 ml of Lysogen broth with 100 μg/ml carbenicillin. Cultures were grown at 37 °C with 200 rpm until an *A*_*600*_ ∼0.6 (600 nm), moved to a room temperature incubator with 200 rpm, and induced (0.1 mM IPTG) for 16 h. Cells were sedimented (10 min; 10,000×*g*), resuspended in 30 ml of lysis buffer (PBS pH 7.2, 1 mM DTT, 1 mM MgCl_2_, 100 μg/ml lysozyme, 1× PIN, and a speck of DNAse1), and rotated at room temperature for 2 h. Cells were passed through a C3 emulsiflex (Avestin) at ∼5 to 10,000 psi three times while on ice, sedimented (10 min; 10,000*g*), and the supernatant was added to 10 ml of glutathione-sepharose beads (10% slurry in PBS pH 7.2, 1 mM DTT) and rotated 4 h at 4 °C. Beads were sedimented (3 min; 500*g*), supernatant was removed, and beads were washed three times for 5 min with 20 ml PBS pH 7.2, 1 mM DTT. For protein elution, washed beads were resuspended in 10 ml PBS pH 7.2 containing 1 mM DTT and 100 μg of TEV protease and rotated overnight at 4 °C. Beads were sedimented (3 min; 500*g*), supernatant containing NanA, NanB, or NanC was retained, concentrated to ∼1 ml using a centrifugal filter (10 kDa MWCO), and the protein concentration was determined with the BCA Protein Assay Kit. Proteins were aliquoted and stored at −80 °C. Densitometry analysis of Coomassie-stained SDS-PAGE gels was performed using ImageJ to determine purity.

### SDS-PAGE

Protein samples were brought up to 10 μl with PBS pH 7.2, mixed with 10 μl of 2× Novex tris-glycine SDS sample buffer containing 0.1 M DTT, heated (95 °C; 5 min), centrifuged (1 min; 10,000*g*), and loaded onto Novex WedgeWell 4 to 12% tris-glycine SDS-PAGE gels. Novex sharp unstained protein standard (5 μl) was included for reference. Gels were run at 150V for 70 min, washed three times with water 5 min, stained using Simple Blue for 1 h, destained overnight in water, and imaged with an Azure C600.

### Neuraminidase activity measurements MUNANA

Activity measurements with MUNANA were performed in 96-well fluorescent assay plates. Samples were brought up to 195 μl with 37 °C reaction buffer (0.1 M KH_2_PO_4_, 1 mM CaCl_2_, pH 6.0), 5 μl of 2 mM MUNANA was added, and the fluorescence (λEx: 355 nm, λEm: 450 nm) at 37 °C was measured (10 min; 15 s intervals) with a Cytation 5 (BioTek) plate reader (74). Activities in relative fluorescence units per second (RFU/sec) were determined by calculating the slope of the early linear region. For Ca^2+^ dependence, samples were brought up in buffer with the indicated CaCl_2_ or EDTA concentration and incubated for 30 min at 37 °C prior to adding MUNANA and taking measurements.

### Size-exclusion chromatography and multi-angle light scattering

SEC was performed with an Agilent 1260 prime high-performance liquid chromatography system equipped with an AdvanceBio SEC 300-Å (7.8 x 300 mm, 2.7 μm) column (Agilent), variable-wavelength detector, and fraction collector. Recombinant proteins and the molecular weight standard (10–20 μl) were injected and run using a 1 ml/min flow rate and PBS pH 7.2 as the mobile phase. Fractions (200 μl) were collected for activity analysis. SEC-MALS was performed on the high-performance liquid chromatography by connecting the variable-wavelength detector to a DAWN8 (Wyatt) and using a 0.3 ml/min flow rate. Molecular weight was calculated with Astra software, version 8.0.1.21. Briefly, peaks were assigned, identical baselines were set for the 280 nm absorbance and 8-angle light scattering chromatograms, and protein concentrations were deduced from the 280 nm using the following molar extinction coefficients (ml∗cm∗mg^-1^): 1.946 (N1), 1.564 (N2), 1.265 (NanA), 1.3 (NanB), and 1.413 (NanC).

### Neuraminidase activity pH optimum determination using MUNANA

Buffers for pH 4, 5, 6, and 7 were prepared by mixing appropriate amounts of 0.1 M citric acid and 0.2 M Na_2_HPO_4_. Buffers for pH 8 and 9 were prepared using 0.1 M NaH_2_PO_4_ and 0.1 M Na_2_CO_3_. All buffers were adjusted to 1 mM CaCl_2_. Standard curves were generated in triplicate for each buffer by mixing 10 μl of 4-MU two-fold serial dilutions (1.5 mM to 732 nM) with 190 μl of buffer and measuring the fluorescence (λEx: 355 nm, λEm: 450 nm) at 37 °C. RFUs were plotted *versus* the 4-MU concentration in the well (Graphpad Prism 8.0), and the linear region slope (RFU/[4-MU]) was used to convert RFU to concentration of cleaved MUNANA for each buffer. To determine the pH optimum activity, the recombinant proteins were diluted in PBS pH 7.2 to a fixed concentration between 2 and 900 nM ([NanC] > [N1] > [N2] > [NanB] > [NanA]). Reaction master mixes were made for each pH buffer by combining 200 μl of 2 mM MUNANA with 3.6 ml buffer and incubated at 37°C. Reactions were performed in triplicate by mixing 10 μl of the diluted protein with 190 μl of the six prewarmed reaction master mixes and the fluorescence (λEx: 355 nm, λEm: 450 nm) at 37 °C was immediately measured. For each protein and pH buffer the slope of the early RFU/sec graph was determined and divided by the pH buffer conversion factor to determine the concentration of cleaved MUNANA/s. The highest activity for each protein was set to 100% and used for normalization.

### Kinetic analysis of neuraminidase activity at different pH values using MUNANA

Recombinant proteins were diluted in PBS pH 7.2 to a fixed concentration between 2 and 900 nM. Two-fold serial dilutions of MUNANA (100 mM to 48.75 μM) were prepared using distilled H_2_O. Reaction master mixes were made for each pH buffer by combining 100 μl of each MUNANA dilution with 1.8 ml of each pH buffer with 1 mM CaCl_2_ and incubated at 37 °C. Reactions for each protein and pH were performed in triplicate by mixing 10 μl of the diluted protein with 190 μl of each prewarmed master mix and the fluorescence (λEx: 355 nm, λEm: 450 nm) at 37 °C was measured. The slope of the early RFU/sec graph was then divided by the pH buffer conversion factor to determine the concentration of cleaved MUNANA/s. Enzyme saturation was confirmed for each sample before performing a Michaelis-Menten kinetic analysis (Graphpad prism 8.0) to calculate *K*_*m*_ and *V*_*max*_ for each protein in each pH buffer. Apparent *k*_*cat*_ values were determined by dividing the *V*_*max*_ by the protein concentration.

### TR1 assay for determining the true k_cat_ for recombinant N1 and N2

Standard curves were generated in triplicate by measuring the fluorescence (λEx: 355 nm, λEm: 450 nm) of F_2_MU in two-fold serial dilutions that ranged from 1 μM to 4 nM at 37 °C for 30 min. Average RFUs were plotted *versus* the F_2_MU concentration in the well (Graphpad Prism 8.0), and the linear region slope (RFU/[ F_2_MU]) was used to convert RFU to concentration of cleaved TR1. TR1 was synthesized as previously described (47, 48). Before use, an equal volume of ethyl acetate was added to TR1, the solution was vortexed, and the top organic layer was removed for a total of 5 times to extract the small amounts of difluoromethylumbelliferyl alcohol that form from spontaneous decomposition. Recombinant neuraminidases were then diluted to ∼0.5 mg/ml and subjected to 5 two-fold serial dilutions in 20 μl of TR1 buffer (50 mM Tris, 20 mM CaCl_2_, pH 7.6). Protein dilutions in triplicate (20 μl each) were incubated 15 min at 37 °C, and each well received 180 μl of master mix containing 2 μl of TR1 and 178 μl of TR1 buffer. The fluorescence (λEx: 355 nm, λEm: 450 nm) at 37 °C was read immediately for 30 min. The fraction of active N1 and N2 was determined by plotting the final concentration of cleaved TR1 (F_2_MU) for each reaction *versus* the protein concentration and determining the slope of the linear correlation plot.

### Thermal and urea stability of the neuraminidases

For thermostability, proteins were diluted at least 40-fold in the different pH buffers containing 1 mM CaCl_2_ and incubated at various temperatures between 37 °C and 95 °C for 10 min in a C1000 Thermal cycler (Bio-Rad). Samples were returned to room temperature and analyzed in triplicate by mixing 10 μl with 190 μl of a 37 °C master mix that consisted of 5 μl of 2 mM MUNANA and 185 μl of the pH buffer containing 1 mM CaCl_2_. The fluorescence (λEx: 355 nm, λEm 450 nm) at 37 °C was read immediately for 10 min, the slope from the early linear graph was recorded, and the slope from the 37 °C heated samples was set to 100% and used for normalization. Curves from the percent activity values were analyzed (GraphPad Prism 8) using a standard curve, sigmoidal, four parameter logistic function with a least squares fit to identify the *T*_*50*_ value which corresponds to the LogIC_50_ (14). For urea stability each pH buffer was adjusted to 4 M urea and 1 mM CaCl_2_. Buffer pHs were corrected, and two-fold serial buffer dilutions were made from 4 M to 0.025 M urea using the same pH buffer. Proteins were diluted at least 20-fold in the pH buffer containing no urea, 10 μl was added to wells containing 185 μl of each pH buffer at the different urea concentrations, and the plate was incubated at room temperature for 30 min. Reactions were initiated by adding 5 μl of 2 mM MUNANA, and the fluorescence was read immediately for 10 min. The data were processed as described above with the slope from the pH buffer containing no urea being used for normalization and the urea concentration in place of temperature.

### Neuraminidase-specific activity measurements using MUNANA and bovine fetuin sialoglycans

An enzyme linked lectin assay (75) was performed in duplicate to assess neuraminidase specific activity on fetuin. Briefly, Maxisorp 96-well plates were coated with 2.5 μg/well of bovine fetuin. Proteins were diluted to equal protein concentrations in reaction buffer (25 mM MES pH 6.5, 150 mM NaCl, 1% BSA, 20 mM CaCl_2_, 0.5% Tween20), two-fold and three-fold serial dilutions were made in the same buffer, and 50 μl of each sample was transferred to the wells of the fetuin-coated plates that already contained 50 μl of reaction buffer in each well. Plates were covered and incubated ∼16 h at 37 °C. Plates were washed six times with PBS pH 7.4 containing 0.05% Tween 20 (PBST) and incubated with 100 μl/well of 1 μg/ml HRP-linked peanut agglutinin for 2 h at room temperature in the dark. Plates were washed three times with PBST and developed with 100 μl/well of 5 μg/ml o-phenylenediamine dihydrochloride prepared in citrate buffer with sodium perborate for 10 min at room temperature. Reactions were stopped with 100 μl/well 1 N sulfuric acid, and the absorbance at 490 nm was measured. The specific activity of the protein dilutions was also measured using MUNANA, where the indicated protein amounts were brought up to 195 μl in 37 °C reaction buffer, 5 μl of 2 mM MUNANA was added, and the fluorescence at 37 °C was monitored for 10 min. Only slopes from dilutions that gave a linear line were included in the graph. For viral neuraminidase comparisons, the proteins and viruses in allantoic fluid were diluted in reaction buffer and standardized by specific activity using MUNANA prior to creating the serial dilutions for the specific activity analysis on fetuin.

## Data availability

All data described are contained within the article, and the raw data are available upon request from the corresponding author R.D.

## Conflict of interest

The authors declare that they have no conflicts of interest with the contents of this article.
